# Studies on the CPA cysteine peptidase in the *Leishmania infantum *genome strain JPCM5

**DOI:** 10.1186/1471-2199-7-42

**Published:** 2006-11-13

**Authors:** Hubert Denise, Jacqueline Poot, Maribel Jiménez, Audrey Ambit, Daland C Herrmann, Arno N Vermeulen, Graham H Coombs, Jeremy C Mottram

**Affiliations:** 1 Wellcome Centre for Molecular Parasitology and Division of Infection & Immunity, Institute of Biomedical and Life Sciences, University of Glasgow, Glasgow, UK; 2 Intervet International B.V., Boxmeer, the Netherlands; 3 WHO Collaborating Centre for Leishmaniasis, Servicio de Parasitología, Instituto de Salud Carlos III, Majadahonda, Madrid, Spain; 4 Division of Cell and Molecular Biology, Imperial College London, London SW7 2AZ, UK

## Abstract

**Background:**

Visceral leishmaniasis caused by members of the *Leishmania donovani *complex is often fatal in the absence of treatment. Research has been hampered by the lack of good laboratory models and tools for genetic manipulation. In this study, we have characterised a *L. infantum *line (JPCM5) that was isolated from a naturally infected dog and then cloned. We found that JPCM5 has attributes that make it an excellent laboratory model; different stages of the parasite life cycle can be studied *in vitro*, it is accessible to genetic manipulation and it has retained its virulence. Furthermore, the *L. infantum *JPCM5 genome has now been fully sequenced.

**Results:**

We have further focused our studies on *LiCPA*, the *L. infantum *homologue to *L. mexicana *cysteine peptidase CPA. LiCPA was found to share a high percentage of amino acid identity with CPA proteins of other *Leishmania *species. Two independent *LiCPA*-deficient promastigote clones (Δ*Licpa*) were generated and their phenotype characterised. In contrast to *L. mexicana CPA*-deficient mutants, both clones of Δ*Licpa *were found to have significantly reduced virulence *in vitro *and *in vivo*. Re-expression of just one *LiCPA *allele (giving Δ*Licpa*::CPA) was sufficient to complement the reduced infectivity of both Δ*Licpa *mutants for human macrophages, which confirms the importance of LiCPA for *L. infantum *virulence. In contrast, *in vivo *experiments did not show any virulence recovery of the re-expressor clone Δ*Licpa*C1::CPA compared with the CPA-deficient mutant Δ*Licpa*C1.

**Conclusion:**

The data suggest that CPA is not essential for replication of *L. infantum *promastigotes, but is important for the host-parasite interaction. Further studies will be necessary to elucidate the precise roles that LiCPA plays and why the re-expression of LiCPA in the Δ*Licpa *mutants complemented the gene deletion phenotype only in *in vitro *and not in *in vivo *infection of hamsters.

## Background

*Leishmania *species are responsible for several pathologies affecting both humans and animals. These parasites are most abundant in developing countries in the Middle East, Asia and South America but also are endemic in the Sub-Mediterranean basin. They have emerged as one of the most important opportunistic agents in AIDS patients [1]. Some cutaneous infections can be resolved and subsequently lead to the development of protection against new infection. These results indicate that immunologic approaches could be effective in preventing or curing infection [2]. However, an effective and safe vaccine has yet to be developed. Efforts towards the development of new cures or vaccines have recently been facilitated by the sequencing of *L. major *[3], *L. braziliensis *and *L. infantum *genomes.

*L. infantum *is a member of the *L. donovani *complex, which is primarily responsible for life-threatening visceral leishmaniasis. These parasites differ significantly from *L. major *and *L. mexicana*, species causing cutaneous leishmaniasis that are classified as Old-world and New-world, respectively, based on their geographical distribution. *L. infantum *is present in both Europe and the Americas, where it has been named *L. chagasi *[4]. Whilst there has been some debate about whether *L. infantum *originated in the Old World or the New World, it has been proposed that *L. infantum *and *L. chagasi *are essentially genetically indistinguishable [5]. *L. infantum *can infect humans and dogs, causing zoonotic visceral leishmaniasis in children and immunosuppressed adults as well as canine visceral leishmaniasis (CanL) [1,4]. CanL is a severe disease that can be fatal if left untreated. Current treatment is complicated and expensive and generally does not result in complete cure. A vaccine would be an important tool in the prevention of CanL and, as dogs are the prime reservoir species for *L. infantum*, could also reduce the incidence of human visceral leishmaniasis [6].

The sequencing projects for *Leishmania *[3] and other trypanosomatids [7,8] have led to the identification of many *Leishmania*-specific genes that potentially might be exploited for vaccine and drug design [9,10]. Moreover, additional candidates are likely to be identified as the currently unidentified gene products are ascribed a function [3]. However, validation or rejection of a protein as a potential vaccine or chemotherapeutic target requires significant experimental investigations that are time-consuming. Thus decisions upon priorities for study are crucial. Investigating the extent to which key proteins are conserved between *Leishmania *species is one primary consideration, as currently it is unclear to what extent knowledge acquired from one *Leishmania *species can be applied to other species. This has important practical applications, as the leishmanias are not all geographically distinct and some virulence factors, when used as a vaccine, have been shown to exhibit distinct protective ability between members of *L. donovani *complex and *L. major *[11-13]. The recent completion of the sequencing of the *L. infantum *and *L. braziliensis *genomes and comparison of these with that of *L. major *has facilitated analyses of gene/protein conservation but also it has revealed *Leishmania *species-specific genes and proteins that could be key mediators of species-specific disease phenotypes.

Cysteine peptidases (CPs) have been characterised as virulence factors and vaccine candidates in *Leishmania *[14-18]. *L. mexicana *possesses three families of related CPs encoded by single (*CPA *and *CPC*) or multicopy (*CPB*) genes [19-21]. While gene deletions of *LmxCPB*, and to a lesser extent *LmxCPC*, lead to an attenuated phenotype, null mutants for *LmxCPA *exhibited no apparent change in phenotype [14]. However, the double null mutant for *LmxCPB *and *LmxCPA *did not induce any lesions in Balb/C mice and the ability of this line to induce an immune response in mice suggested that it has potential as an attenuated live vaccine [14,22].

In order to assess the level of conservation in both structure and function of these CPs between *Leishmania *species, we have initiated a characterisation of cysteine peptidase orthologues in a cloned *L. infantum *line (JPCM5) derived from a recent isolate from a naturally infected dog in Spain. To validate the usefulness of JPCM5 for this and other experimental studies, we have tested whether the clone has the essential characteristics for a laboratory model: that it causes visceral leishmaniasis in experimental animals and is accessible to most experimental studies including reproduction of its life cycle *in vitro *and genetic manipulation. We present here the results of this analysis and the use of the clone for a molecular study on *LiCPA*.

## Results

### Isolation and characterisation of L. infantum JPCM5

*Leishmania *parasites were recovered from a naturally infected dog from the Madrid area, Spain. They were characterised as *L. infantum *zymodeme 1 (MON-1) by isoenzyme analysis [23] and transformed into promastigotes *in vitro *before being stored under liquid nitrogen as JPC (MCAN/ES/98/LLM-724). Five independent clones (JPCM1 to JPCM5) were derived from this stock by colony cloning and tested for infectivity in hamsters. Two of the cell lines were recovered from the spleen 15 weeks after infection confirming that they had conserved their infection potential. Clone M5 (JPCM5; MCAN/ES/98/LLM-877) was selected for all further studies.

JPCM5 grew well as promastigotes *in vitro *in many of the media used to grow *Leishmania*, including RPMI, SDM79 and NNN. A temporary increase of serum concentration from 10 to 20% (v/v) promoted the parasite's growth following adaptation to a different medium, cloning, transformation from, or to, amastigotes and transfection experiments. In HOMEM medium with 10% (v/v) serum, the parasites had a doubling time of 16.0 ± 2.7 h. After 4 to 5 days in culture, when stationary phase had been reached, most promastigotes had a metacyclic morphology, indicating that metacyclogenesis had occurred. Stationary phase promastigotes of JPCM5, grown in HOMEM medium, were transformed *in vitro *into amastigote-like forms using conditions previously described for other *L. infantum *lines [24]. The transformation of the entire culture, according to morphological parameters, required 3 to 4 days at 37°C in acidic medium (pH 5.5 for SDM or pH 6.5 for MAA). This was accompanied by changes in protein profile as determined by SDS-PAGE (data not shown). The axenic amastigotes could be maintained, with the help of regular sub-passages, or transformed back into promastigotes by switching the culture conditions to 28°C in HOMEM medium with 10% (v/v) FCS. It generally required four days for transformation fully back to promastigotes.

*L. infantum *JPCM5 were infective to human and dog macrophages (see below) and hamsters. JPCM5 parasites could be recovered from spleen, liver and bone marrow of infected hamsters less than two months post infection. Long-term *in vitro *culture of some *Leishmania *species has been reported to result in loss of virulence [25]. To test whether this applied to JPCM5, promastigotes were sub-passaged *in vitro *for 8, 17 and 25 weeks (corresponding to about 10, 20 and 30 sub-passages, respectively) before being inoculated into hamsters. In each case, amastigotes were recovered from the spleen after less than three months indicating that the promastigotes had not lost their virulence and were still able to establish an infection. These *in vitro *and *in vivo *results indicated that JPCM5 is suitable for most cell-culture studies.

### Isolation of the CPA cysteine peptidase gene

**Figure 1:**
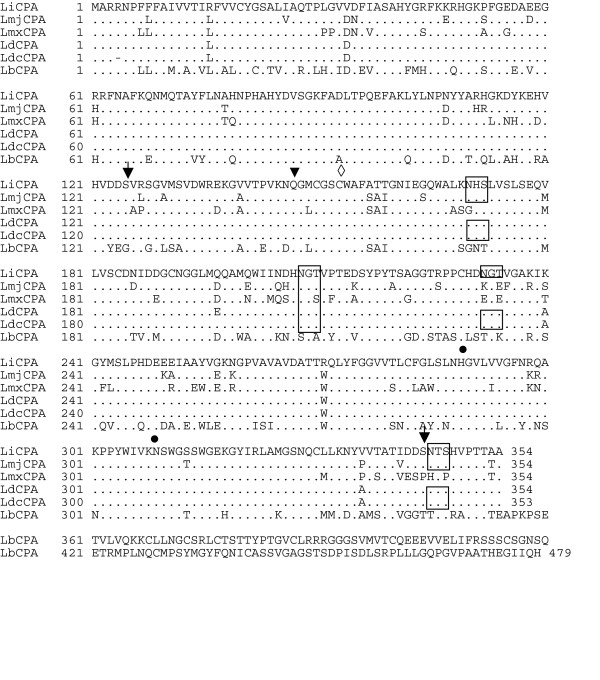
**Alignment of CPA amino acid sequences**. Alignment of deduced amino acid sequence of *LiCPA *(CAD12932) from *L. infantum *JPCM5, with deduced amino acid sequences obtained from GenBank. These included *L. majo*r (*LmjCPA*, CAC18865.1), *L. mexicana *(*lmxCPA*, CAA44094), *L. donovani *(*LdCPA*, AAK27384.1), *L. chagasi *(*LdcCPA*, AAC38833) and *L. braziliensis *(*LbCPA*, LbrM19.1180). The dots represent amino acid identity. The arrows show the predicted cleavage sites of pro/mature domains and mature/C-terminal extension, respectively. Amino acid residues important for the catalysis are indicated as follows : active site cysteine C^153 ^(◇), the predicted catalytic dyads H^289 ^and N^309 ^(●) and the predicted glutamine of the oxyanion hole Q^147 ^(▼). The potential glycosylation sites are shown in boxes.

The complete *CPA *gene of *L. infantum *JPCM5 (*LiCPA*), including the ORF and the 5' and 3' flanks, was amplified by PCR using primers derived from the *L. mexicana CPA *[21] and sequenced. The *LiCPA *ORF sequence predicted a protein of 354 amino acids corresponding to a size of 38.9 kDa, an isoelectric point of 7.6 and possessing a 10 amino acid C-terminal extension similar to the *L. mexicana CPA *[21]. Comparison of the deduced amino acid sequence with the translated *CPA *sequences from several *Leishmania *species is shown in Figure 1. LiCPA shares 97.7% amino acid identity with the published *L. chagasi *CPA (LcCPA) [26], 87.3% with *L. major *CPA (LmjCPA) and 80.3% with *L. mexicana *CPA (LmxCPA) protein sequences, respectively. There are three potential N-glycosylation sites in LiCPA, two in the mature domain (^208^NGT^210 ^and ^170^NHS^172^) and one at the beginning of the C-terminal extension (^345^NTS^347^); the last two being conserved in all sequences except in *L. mexicana *and *L. braziliensis*. However, amino-acid residues important for catalysis, ^153^C, ^289^H and ^309^N, as well as the predicted glutamine of the oxyanion hole ^147^Q, are conserved in all sequences [21]. LiCPA is identical in size to CPA from *L. major*, *L. mexicana *and *L. donovani*, but differs from LbCPA, which has a 123 amino acid C-terminal extension. A C-terminal extension is characteristic of the cathepsin L-like CPB cysteine peptidases of *Leishmania *and other trypanosomatids [20,27], but has not been observed before in any CPA protein.

**Figure 2:**
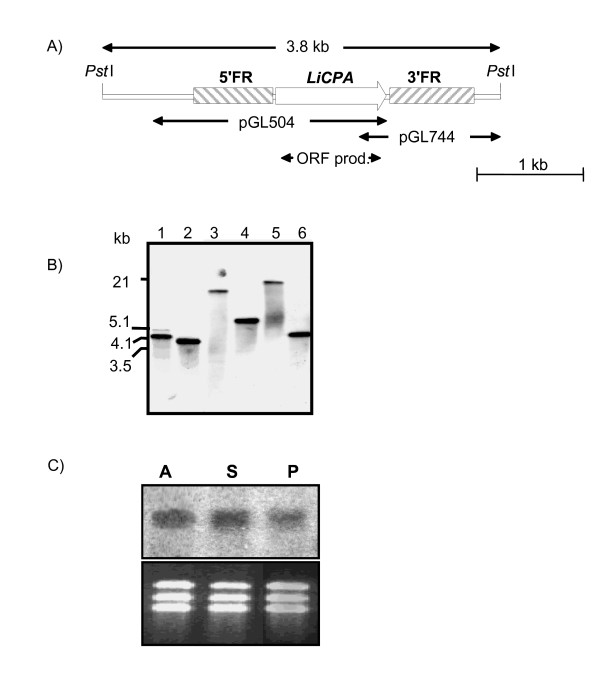
***LiCPA *genomic organisation and expression**. A) Map of *LiCPA *locus with the different subclones analysed indicated. B) Southern blot of *L. infantum *JPCM5 gDNA hybridized with the *LiCPA *PCR product. The gDNA was digested using *Sph*I (lane 1), *Pst*I (lane 2), *Xho*I (lane 3), *Sma*I (lane 4), *Sac*I (lane 5) and *Apa*I (lane 6). C) Northern blot of total RNAs from *L.infantum JPCM5 *log phase promastigotes (P), stationary phase promastigotes (S) and axenic amastigotes (A) hybridized with the *LiCPA *ORF PCR product. After washing, the membrane was exposed on a phosphor-activated screen. An ethidium bromide-stained gel of RNA samples for each life cycle stage is shown in the lower panel.

**Figure 3:**
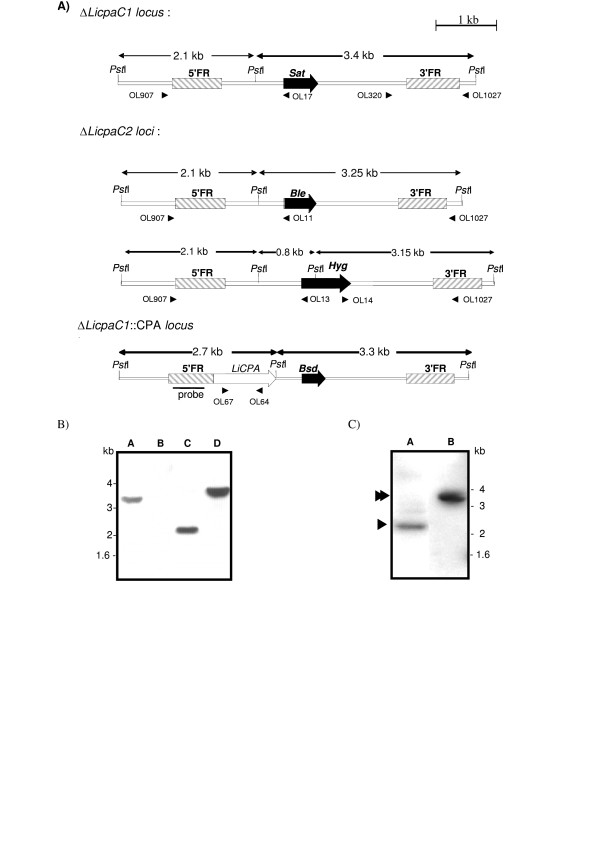
**Generation of *Licpa *null mutants**. A) *Pst*I restriction map of Δ*Licpa*C1, Δ*Licpa*C2 and Δ*Licpa*C1::CPA loci. The size of the restriction fragments expected is indicated. 5'FR and 3'FR indicate the 5' and 3' *LiCPA *flank sequence, respectively. The arrowheads indicate the position of the different primers used to confirm the correct integration of the disruption constructs by PCR. B) Southern blot analysis of the Δ*Licpa*C1 mutant DNA digested with *Pst*I and hybridized with the following probes : *LiCPA *3'FR (lane A), ORF (lane B) and 5'FR (lane C). Lane D shows *L. infantum *DNA hybridized with the 3'FR probe. C) Southern blot analysis of Δ*Licpa*C2 mutant (lane A) and wild type (lane B) DNA hybridized with the *LiCPA *5'FR probe. The single and double arrowheads indicate the deleted and the wild-type alleles, respectively.

**Figure 4:**
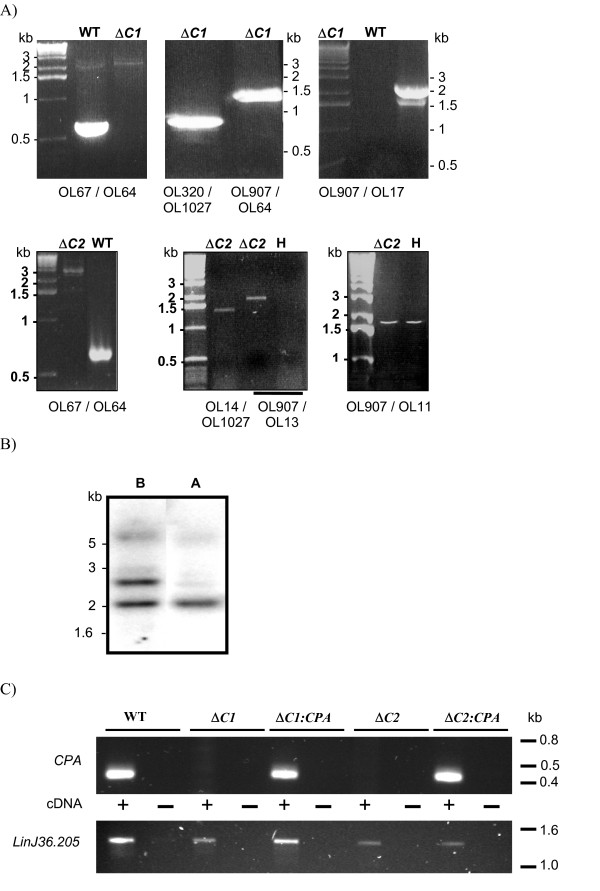
**Analysis of *Licpa *null mutants**. A) PCR analysis of the two Δ*Licpa *mutants. PCR reactions were performed using primers indicated in Figure 3A. WT: wild-type, H; heterozygote *CPA*/Δ*Licpa*, Δ*C1*: Δ*LicpaC1 *and ΔC2: Δ*Licp*aC2. B) Southern blot analysis of the Δ*Licpa*C1 (lane A) and Δ*Licpa*C1*::CPA *(lane B) re-expresser mutant DNA digested with *Pst*I and hybridized with a 5'FR probe. C) RT-PCR analysis of wild type (WT), Δ*LicpaC1 *(Δ*C1*), Δ*Licp*aC1*::CPA *(Δ*C1:CPA*), Δ*LicpaC2 *(Δ*C2*) and Δ*Licp*aC2*::CPA *(Δ*C2:CPA*) parasites. Nested PCR were carried out in presence (+) or in absence (-) of cDNA using the primer pairs OL136/SL primer. Lower panel is a control with the *LinJ36.2050 *gene.

Southern blot analysis showed that *LiCPA *is single-copy (Figure 2, panels A and B) and Northern blotting showed that *LiCPA *is expressed at similar levels in log phase and stationary phase promastigotes and also amastigotes (Figure 2C). A *LiCPA *deletion construct containing the *SAT *gene flanked by about 800 bp of *LiCPA *5' and 3' flank regions was generated and used to transfect JPCM5 parasites. The parasites were selected in 96-well plates with HOMEM medium supplemented with the antibiotic nourseothricin. In these conditions, one clone was obtained seven weeks post-transfection. Southern blot analysis (Figure 3B) and PCR analyses (Figure 4A) showed correct integration of the *SAT *construct into the *LiCPA *locus. Moreover, the wild type alleles could not be amplified with *CPA *gene specific primers (Figure 4A), and *CPA *mRNA could not be detected (Figure 4C), confirming that this nourseothricin-resistant clone (named Δ*Licpa*C1) is a null mutant resulting from loss of heterozygocity.

A second transfection was performed on JPCM5 promastigotes with a second deletion construct derived from the previous one by replacement of the *SAT *marker by the *BLE *gene. The transfection and selection steps were performed as previously, except that 10 μg/ml of phleomycin was used in place of nourseothricin. Four clones grew onto agar plates after two weeks. PCR (Figure 4A) and Southern blot (not shown) analysis demonstrated that these four clones were Δ*Licpa *heterozygote mutants having correctly integrated the *BLE *construct into one *LiCPA *allele. One of these (clone 3047) was selected and re-transfected with a third *CPA *knockout construct, pGL813, carrying the *HYG *gene. A total of 16 clones were obtained from four 96-well plates after two weeks of selection in the presence of both phleomycin and hygromycin. Southern analysis of one of these clones, called Δ*Licpa*C2, confirmed that it was indeed a hygromycin- and bleomycin-resistant Δ*Licpa *null mutant (Figure 3C) and RT-PCR analysis of cDNA showed the absence of *CPA *mRNA (Figure 4C). The *LiCPA *gene was re-introduced into the CPA locus in both Δ*Licpa *mutants. The re-integration construct, pGL793, contained the *LiCPA *ORF surrounded by the native 5' flank with 3' sequence derived from the *DHFR *locus, and the blasticidin-resistance gene enclosed between the 5' and 3' *DHFR *flank sequences. Both Δ*Licpa *mutants were transfected with the re-integration cassette and selection achieved in the presence of 10 μg/ml of blasticidin. Several clones were isolated from each transfection (the ones characterised in this study were named Δ*Licpa*C1*::CPA *andΔ*Licpa*C2*::CPA*) and the correct replacement of previously integrated deletion cassettes was confirmed by PCR (Figure 4A) and Southern blot (Figure 4B). *LiCPA *re-expression in Δ*Licpa*C1 and Δ*Licpa*C2 was demonstrated by RT-PCR (Figure 4C).

**Figure 5:**
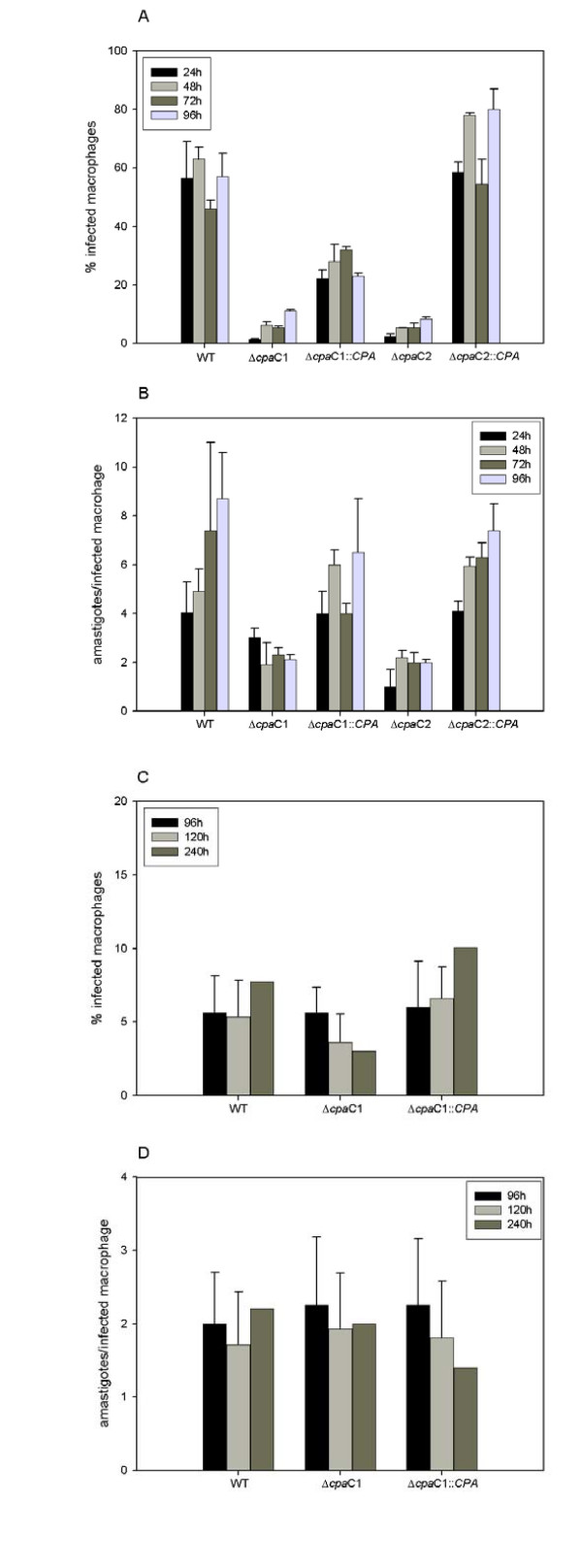
***LiCPA *mutant infectivity *in vitro***. Human monocyte cells (U937) were infected with wildtype (WT), CPA null mutants (Δ*Licpa*C1, Δ*Licpa*C2) or re-expresser clones (Δ*Licpa*C1::*CPA*, Δ*Licpa*C2::*CPA*) of *L. infantum*. Cell cultures were monitored for four days; results are presented as the percentage of infected macrophages and the number of amastigotes per infected cell in panels A and B, respectively. Similar experiments were performed in a canine monocyte cell line (DH82) using WT, Δ*Licpa*C1 and Δ*Licpa*C1::*CPA L. infantum *clones. Cell cultures were monitored for five to ten days after infection; results are presented in panels C and D. Depicted values at 96 and 120 hrs incubation are the mean of four independent experiments and error bars indicate the standard deviation. The data for the ten day incubation period is derived from one experiment, hence the absence of a standard deviation.

The Δ*Licpa*C1 and Δ*Licpa*C2 mutants were grown as promastigotes in HOMEM medium. In these conditions, they exhibited no apparent growth reduction compared with wild type JPCM5. They were also both able to transform into axenic amastigotes. In order to assess whether the mutants lacking CPA were virulent, their infectivity toward human macrophages was investigated. In comparison with JPCM5, the Δ*Licpa*C1 and Δ*Licpa*C2 mutants had a reduced ability to infect human macrophages (Figure 5A) and a reduced number of amastigotes/infected macrophage (Figure 5B). The infectivity was restored to wild type levels by re-expression of *CPA *in the Δ*Licpa*C2 mutant, but Δ*Licpa*C1*::CPA *had a virulence phenotype midway between the Δ*Licpa*C1 mutant and wild type parasites. Some cell lines were also tested for their ability to infect macrophages of dogs (Figure 5C and 5D). No statistically significant differences (ANOVA; p ≤ 0.05) were observed between wild type, Δ*Licpa*C1 orΔ*Licpa*C1*::CPA *with respect to percentage of infected macrophages or the number of amastigotes per infected macrophage.

**Figure 6:**
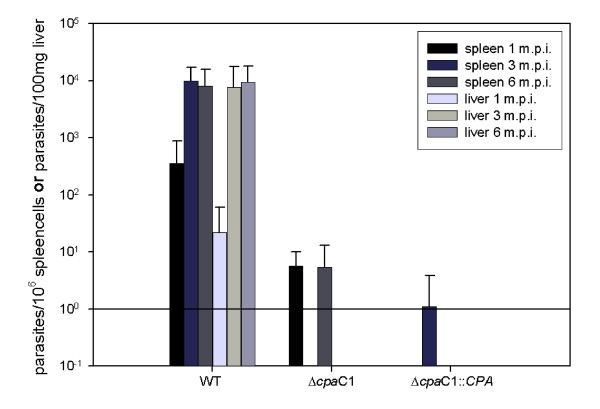
***LiCPA *mutant infectivity *in vivo***. Hamsters were infected with wild type (WT), CPA null mutant (Δ*Licpa*C1) or re-expresser clone (Δ*Licpa*C1::*CPA*) of *L. infantum*. The parasite burden in spleen and liver was determined by limiting dilution culturing at one, three and six months post infection (1, 3 or 6 m.p.i.). Results are shown as the average parasite burden per group; error bars indicate the standard deviation.

The virulence of Δ*Licpa*C1 to hamsters was found to be significantly less than for the wild type JPCM5. In a hamster infection model (Figure 6) parasites were not detected in the liver and only very low levels were detected in the spleen, with the number of parasites decreasing between 1 and 6 months post-infection. The overall parasite burden for these clones was between 10^2^- and 10^4^-fold lower compared with JPCM5 at 1 and 6 months post-infection, respectively. The virulence of Δ*Licpa*C1*::CPA *was also compared *in vivo *to JPCM5. Low numbers of parasites were found in the spleen of Δ*Licpa*C1*::CPA *infected hamsters 3 months post-infection, whereas no parasites were detected in spleen or liver of hamsters at other time points, indicating the virulence was not restored in the re-expresser clones.

## Discussion

*L. major *was the first *Leishmania *species to have its genome sequenced [3]. For comparative genomics, it was thought that the second *Leishmania *species to have its genome sequenced should be a causative agent of visceral leishmaniasis. There are several closely-related species of the *L. donovani *species complex that are found in different geographical regions. *L. donovani *is the primary cause of visceral leishmaniasis in the Indian subcontinent and East Africa, *L. infantum *in the Mediterranean region and *L. chagasi *in the New World. Humans are the only known reservoir of *L. donovani *in India, while canines, especially domestic and stray dogs, provide the main reservoir host for *L. infantum *and *L. chagasi *[4]. While the last two species are considered to be genetically identical [4], all three are very similar. Based on gene sequencing and microsatellite analysis (the most sensitive method currently available for discriminating between populations), recent studies have been unable to separate East African *L. donovani *strains from *L. infantum *[28,29]. These African strains are also very close to the Indian *L. donovani *strains. Thus, it could be argued that whichever *L. donovani *complex species was chosen for the genome project, the nucleotide sequence data generated from the protein coding regions would be likely to be almost identical. However, in order to maximise the value arising from the genome sequencing, the parasite needs to be an experimentally amenable organism.

The data resulting from the analyses described in this paper show that *L. infantum *JPCM5 has the following characteristics that make it a suitable line for many experimental studies. It has been characterized as belonging to zymodeme MON-1 by isoenzyme typing. MON-1 is one of the most prevalent zymodemes in Europe and the Mediterranean basin, in countries such as Greece, France, Spain, Italy, Algeria and Syria, and is also found in Brazil [30-35]. JPCM5 has been shown to be virulent to hamsters (this study) and dogs [36], causing the symptoms of visceral leishmaniasis in both animals. These observations are important as they show that the parasite has maintained its virulence following isolation, cloning and prolonged culture in the laboratory. The three major life-cycle stages of JPCM5 can be grown *in vitro*. Importantly, amastigotes can be propagated *in vitro*, and grown in canine macrophages or human macrophages. We have also shown that JPCM5 can be transfected successfully with both integration (this study) and episomal (unpublished) vectors. Transfected mutants can be cloned on soft agar plates or by limiting dilution. The strain is therefore suitable for genetic manipulation studies on genes identified from the genome project. JPCM5 maintains virulence to animals after transfection and cell culture (> 2 months). This is important as it allows virulence studies to be carried out on mutant parasites defective for virulence genes. Many isolates of *L. donovani *lose their virulence after transfection and prolonged culture, making it difficult to use this species for molecular genetic studies on virulence [25], thus the suitability of JPCM5 for such studies is an important attribute. *L. infantum *JPC is infective for sandfly vectors of the species *Lutzomyia longipalpis *[37], one of the most widely available laboratory colonized sandfly [38]. Finally, the *L. infantum *JPC isolate can be successfully used as a challenge strain for vaccine studies [36].

This study has confirmed through analysis of the CP CPA that *L. infantum *JPCM5 is experimentally useful. Moreover, the investigation has provided important insight into how the roles of proteins may differ between *Leishmania *species. Our laboratory previously demonstrated that cysteine peptidases are potent virulence factors in *L. mexicana *[14,16]. Whilst *L. mexicana *Δ*cpa *mutants did not show a defect in virulence *in vivo *[39], the Δ*cpa/cpb *double mutants had a more defined virulence phenotype than Δ*cpb *– indicating that CPA plays an important role in this parasite [14,22]. Virulence determinants are not always conserved between species; for example the *L. donovani *major antigen A2 was shown to not be expressed in *L. major *[40]. Hence, we undertook the study of LiCPA to determine how similar its role is in *L. infantum *and *L. mexicana*.

We deleted *CPA *in *L. infantum *and tested the mutants for virulence *in vitro *and *in vivo*. Two independent Δ*Licpa *mutants were characterised in this study. The first mutant generated, Δ*LicpaC1*, was the result of a loss of heterozygocity, an event that has been reported to occur naturally in response to an increase of drug concentration and involves duplication of the allele carrying the antibiotic resistance gene which leads to higher expression of the gene product essential for parasite survival [41]. The second mutant, Δ*LicpaC2*, was generated classically using two independent markers and methods identical to the ones used for *L. mexicana *[42]. For both mutants, molecular analysis confirmed that the expected gene replacements had taken place, so the Δ*Licpa *mutants could be considered genetically indistinguishable at the level of the deleted *LiCPA *locus. Indeed both mutants exhibited an attenuated phenotype, *in vitro*, in human macrophages, and, *in vivo*, in hamster infections. This indicates that LiCPA might play a more important role in *L. infantum *virulence than was found in the *L. mexicana *studies. These findings were apparently not corroborated by dog macrophage experiments where Δ*Licpa*C1 was found as infective as wild type parasites. However, the overall level of infection achieved – almost 12 times fewer dog macrophages were infected than human macrophages with JPCM5 wild-type parasites – may have been too low to truly analyse the phenotype of Δ*Licpa*C1. On the other hand, the number of amastigotes per infected macrophage was not significantly different between wild type and mutant parasites, which suggests that LiCPA is not required for *L. infantum *survival in dog macrophages. One possible explanation for the different behaviour of the parasites in human and canine macrophage cell lines is the different status of the host cells: the U937 human monocytes were activated by PMA, whereas the DH82 canine monocytes were not activated prior to addition of the parasites. The low percentage of infection of the DH82 cells maybe due to the non-activated status of the monocytes and consequently differences in virulence between the parasite lines would be harder to detect in such a system.

The finding that re-expression of only one *LiCPA *gene was sufficient in itself to complement the reduced infectivity of both *LiCPA-*deficient mutants for human macrophages confirm the importance of LiCPA for *L. infantum *virulence to these cells. Δ*Licpa*C1::CPA was only half as virulent as Δ*Licpa*C2::CPA, this may reflect a lower level of LiCPA re-expression in the first cell line. Alternatively, we could not exclude that the loss of heterozygocity had not been accompanied by other re-arrangements destined to help the parasites to adapt to the higher drug concentration and consequently that Δ*Licpa*C1 and Δ*Licpa*C1::CPA were less fit than Δ*Licpa*C2 and Δ*Licpa*C2::CPA to infect macrophages; investigating this will require more sensitive methods as the level of macrophages infected observed with the deficient mutants were very low. In contrast, *in vivo *experiments did not show any recovery in virulence of Δ*Licpa*C1::CPA compared with Δ*Licpa*C1. This is not very different from the results in human macrophages since here the virulence of the Δ*cpa*C1 mutants was also found to be strongly reduced while the virulence was only partly restored in Δ*cpa*C1::*CPA *(in contrast to Δ*cpa*C2::*CPA*). One explanation is that the Δ*licpa*C1::CPA clone expresses only one *LiCPA *allele whereas wild type parasites have two. Also there is dissimilarity between the *LiCPA *genetic context in these two cell lines; the 5' *LiCPA *flank sequence of both clones was identical, however the nature of the 3' flank was different in that the re-expressers possessed the *L. major *3' DHFR flank sequence downstream of the *LiCPA *gene. Therefore the level of expression of CPA may not precisely mimic the *in vivo *situation during the infection process and during differentiation of promastigote to amastigote. The DHFR sequence is classically used in *Leishmania *constructs to stabilize the mRNA, leading to higher levels of expression [43,44]. The exact nature of these key signals is still cryptic and the re-expresser lines may provide a tool to investigate them.

The results of this study differ in several key respects to those of Mundodi et al., who described the characterisation of the *Lccys2 *gene in *L. chagasi *[45]. *Lccys2 *[26] is the functional and syntenic homologue of *L. infantum *CPA and differs in sequence at only 6 amino acid positions. *Lccys2 *expression is amastigote-specific, yet surprisingly Mundodi et al. were unable to obtain homozygous null mutants of *Lccys2 *in the promastigote life-cycle stage [45]. In contrast, in this study we showed that LiCPA is expressed in all life-cycle stages analysed and we were able to generate several independent *LiCPA *null mutants, which were viable as promastigotes *in vitro*. The efficiency of transformation can vary between strains of *Leishmania *and between species, which might account for the differences observed in the ability to isolate genetically modified parasites. Indeed, our studies indicate that *L. chagasi *and *L. infantum *may not be genetically indistinguishable, as has been proposed [5], but the two species might have species-specific differences with functional consequences. It is possible that there have been genome re-arrangements to compensate for the loss of *CPA*, but these would be hard to detect. There is likely to be some plasticity in the genome of different strains of *L. infantum*, but JPCM5 is a good starting point on which to base further studies on genetic variation between isolates of the same species. However, both this study and that of Mundodi et al. identified the importance of the *LiCPA*/*Lccys2 *gene in *Leishmania infantum/chagasi *infection and pathogenesis.

## Conclusion

We provide evidence that the CPA cysteine peptidase is not essential for replication of *L. infantum *promastigotes, but is important for the host-parasite interaction. Determining the precise roles that LiCPA plays in the intracellular amastigote stage of the parasite will require further investigation.

## Methods

### Parasite lines

*L. infantum *JPC (MCAN/ES/98/LLM-724) was isolated in the WHO Collaborating Centre for Leishmaniasis, ISCIII, Madrid, Spain from the spleen of a naturally-infected dog residing in the area in 1998. The parasites were maintained in Novy-MacNeal-Nicolle (NNN) medium (66% (v/v) bacto-agar, 33% (v/v) defibrinated rabbit blood) at 28°C. The resulting promastigotes were grown in RPMI medium before cloning by limiting dilution in agar blood plates. Five clones were isolated, JPCM1–JPCM5, but all further analyses were carried out with JPCM5 (MCAN/ES/98/LLM-877).

### Parasite in vitro culture

JPC and JPCM5 promastigotes were grown in modified Eagle's medium (designated HOMEM medium, RPMI, SDM79 or NNN media with 10% (v/v) heat-inactivated foetal calf serum (FCS) at 28°C. Changes between media were accompanied by temporary elevation of FCS concentration to 20%(v/v). Cultures were sub-passaged to 0.5–1 × 10^6 ^cells/ml in fresh medium every 3–4 days when the culture had reached late logarithmic phase. The exponential curve regression, the specific growth rate and the doubling time were calculated using Microsoft Excel software from four independent experiments.

Axenic cultures of amastigote-like forms were performed in MAA medium following the protocol previously published with minor modifications [24]. Briefly, promastigotes were grown for 4 to 5 days in HOMEM medium supplemented with 10% (v/v) FCS and 24.5 mM hemin. Then 1–5 × 10^7 ^parasites were pelleted, washed once in PBS, re-pelleted and re-suspended in 5 ml of MAA2 medium (modified medium 199 with Hank's salts (Gibco BRL), 0.5% soybean trypto-casein (Promega), 20% (v/v) FCS, 4.8 mM L-glutamine, 24.5 mM hemin, 4 mM NaHCO_3 _and 25 mM HEPES pH 6.5). The parasites were then incubated at 37°C in the presence of 5% CO_2 _for 2 to 3 days before the medium was first changed. Thereafter the medium was changed every 4 to 5 days.

Transformation from amastigotes to promastigotes was performed by transferring 10^6^–10^7 ^amastigotes, which were washed once in PBS, into 10 ml of HOMEM medium supplemented with 20% (v/v) FCS. After incubation at 28°C under air until more than 90% of the parasites were flagellated (generally after 4 to 5 days), the medium was changed to HOMEM supplemented with 10% (v/v) FCS.

### Parasite transfections and selection

Transfections were performed as previously reported [46] using 20 μg of linear DNA. 24 h after the transfections, mutant parasites were selected using one or more antibiotics: 20 μg/ml nourseothricin (Hans Knoll Institute, Germany), 50 μg/ml hygromycin B (Roche, Germany), 10 μg/ml phleomycin (Cayla, France) and/or 20 μg/ml blasticidin (Cayla, France). Clones were either obtained on 0.7% agar-HOMEM plates or in liquid medium, in presence of the appropriate antibiotics, and propagated in HOMEM medium supplemented with the same antibiotics.

### Macrophage infections

The U937 human monocyte line, which is non-adherent, was differentiated to macrophages in RPMI with 5% (v/v) FCS supplemented with 0.1 ng/ml phorbol 12-myristate 13-acetate (PMA) at 37°C in presence of 5% CO_2_. Differentiation had occurred by 72 h when the cell line was adherent. The cells were washed with RPMI to remove non-adherent cells before adding the JPCM5 parasites at a ratio of 1:20 (macrophages:*Leishmania*). The infected cultures were incubated at 37°C in the presence of 5% CO_2_. After 3 hours the cultures were washed to remove free *Leishmania*. Samples of infected macrophages were fixed and stained with Giemsa stain at 24, 48, 72 and 96 h post-infection. For each time point, at least 100 macrophages were observed and the number of amastigotes per infected cell was counted. The results were expressed in terms of the % infected macrophages and the number of amastigotes/infected macrophage.

Infections of the canine monocyte-macrophage cell-line DH82 were performed essentially as described by Kiderlen and Kaye [47]. Briefly, adherent and non-adherent DH82 cells were collected in polypropylene round-bottom tubes and stationary phase promastigotes were added at an effector-to-target ratio of 1:8. The cell suspension was incubated for 2 hours at 37°C with gentle agitation. Remaining extra cellular parasites were washed off by differential centrifugation (220 × g, 8 min, 4°C, four repeats); the resulting cell suspension was seeded into 4-chamber LabTek tissue culture slides (Nunc, Denmark) and incubated for 120 hours. Slides were stained with Giemsa solution (Merck, Germany). For each time point at least 400 macrophages were observed and the number of amastigotes per infected cell was counted; results were expressed as the % infected macrophages and the number of amastigotes/infected macrophage.

### Hamster infections

Infections of Golden Syrian hamsters were initiated by intraperitoneal (i.p.) inoculation with 10^7 ^stationary phase promastigotes in PBS. The hamsters' weight was monitored every week. The hamsters were sacrificed at 3, 6 and 9 months post-infection and the spleens were weighed and sliced into pieces to collect amastigote parasites. These were transformed *in vitro *at 28°C in HOMEM medium supplemented with 20% (v/v) FCS to establish promastigote cultures.

Hamsters used for the study of parasite virulence were inoculated i.p. with 10^8 ^stationary phase promastigotes and sacrificed at 1, 3 and 6 months post-infection. Spleen and liver samples were used for parasite quantification by culture microtitration [48].

### PCR amplification

Genomic DNA was prepared from large scale promastigote cultures and extracted using phenol as previously published [21]. PCR was carried out using 50 ng of genomic DNA as template, 100 ng (about 30 pmoles) of each primer, 5% DMSO and 2.5 U Taq polymerase (Perkin-Elmer). Conditions were 1 cycle of 94°C for 5 min then 25 cycles of 94°C for 1 min, 60°C for 2 min and 72°C for 2 min followed by 1 cycle of 72°C for 5 min.

### LiCPA deletion and re-expression constructs

JPCM5 *CPA *flanking regions were amplified from *L. infantum *DNA with primers OL670–OL671 and OL672–OL673 originally designed to amplify *L. mexicana *sequences and integrated in place of the *L. mexicana *flank sequences in each side of the *DHFR-SAT *cassette of the *Lmxcpb-sat *deletion construct [22] to generate pGL545. The *LiCPA BLE *(pGL726) and *HYG *(pGL813) deletion constructs were derived from pGL545 by replacing the *Spe*I/*Bam*HI fragment containing the *SAT *resistance gene by the appropriate antibiotic resistance gene extracted from pGL53 and pGL345 (p *Lmxcpb-ble *and p *Lmxcpb-hyg *constructs), respectively. A 1.55 kb DNA fragment corresponding to the *LiCPA *5' flank and ORF was amplified with primers OL1005 and OL1006 in order to generate *Sac*II and *Pst*I sites for sub-cloning. The *LmxCPC *5' and 3' flank sequences from pGL545 [49] were then replaced by this PCR product and the *LiCPA *3' flank, amplified as described above, to generate the *LiCPA *re-expression construct pGL793.

### Southern blot analysis

For Southern blot analysis, 5 μg *L. infantum *genomic DNA was digested to completion with the appropriate restriction enzymes before being separated and transferred onto Nylon membrane as described previously [22]. For wild-type *L. infantum *DNA analysis, the membrane was treated and probed with a non-radioactively labelled PCR product corresponding to the *LiCPA *ORF as described above. Δ*Licpa *mutant blots were probed with radioactively labelled PCR products corresponding to the *LiCPA *ORF 5' or 3' flanks, essentially as published [50] except that the membranes, after high stringency washes, were exposed on Phosphorimager plates. The plates were scanned at high resolution on a Typhoon 8600 apparatus (Molecular Dynamics).

### Northern blot analysis

Total RNA was extracted from 10^7 ^to 10^8 ^parasites using Trizol (Invitrogen, UK) or RNAEasy kit (Qiagen, Germany) according to the manufacturer's instructions. The amount of total RNA extracted was quantified by spectrophotometry and 5 μg were loaded on 0.8% agarose gel prepared in DEPC-treated 0.5× TBE buffer. After separation, gels were washed once in DEPC-water and transferred onto membrane using to the same procedure as for Southern blot. Hybridization with radioactively labelled probes took place overnight at 65°C. Stringent washes were performed in 0.2× SSC, 0.1% SDS and the membranes were exposed onto Phosphorimager plates. The plates were scanned at high resolution after two days exposure.

### RT-PCR

cDNA was prepared from 5 μg of total RNA using the AMV RT module from the GeneRacer kit (Invitrogen, UK). A first RT-PCR was performed on 1 μl of cDNA mixture with 2 units of Thermozyme enzyme (Invitrogen, UK) and 100 ng of first-round primers in 50 μl reaction volume. The resulting PCR mixture was diluted 30 times in water and 1 μl of it was used as template for a nested reaction performed in a 20 μl reaction volume with 1 unit of *Taq*DNA polymerase (ABGene, UK) and 100 ng of nested primers. The first-round primers used were the Splice Leader primer (OL618)/OL137 for the 5' amplification, and OL67/GeneRacer Oligo dT primer for the 3' amplification. The primers for the nested PCR were OL618/OL136 for the 5' amplification and OL1194/GeneRace 3' Nested primer for the 3' amplification. OL1984 and OL1985 were used to PCR amplify *LinJ36.2050*.

Oligonucleotides used in this study.

OL67 5'-CAGAACATGCAGACAGCC-3'

OL136 5'-GAGCGGAACCGTAGCACA-3'

OL137 5'-GACAGCGTCCGCAGTGGTG-3'

OL618 5'-AAGTATCAGTTTCTGTACTTTATG-3'

OL670 5'-ATATAAGCTTCTACTGCACCAGGTACTG-3'

OL671 5'-ACGTGTCGACGAGAAGGACGTGACGGGG-3'

OL672 5'-GGTGCCCGGGGCTGTGCACAAACACGACC-3'

OL673 5'-GTCGAGATCTCACTGTCCATGGCACGCCTG-3'

OL1006 5'-CTGCAGCTAGGCCGCTGTCGTCGG-3'

OL1194 5'-GACAGCGTCCGCAGTGGTG-3'

OL1984 5'-GCACCCGGGATGGCGGCAACTCATCTTAC-3'

OL1985 5'-GCAGGATCCTCACTTCGGCAAACCGTTCTTTC-3'

## Abbreviations

LiCPA : *L. infantum *CPA protein (similar for *L. major *(LmjCPA); *L. mexicana *(LmxCPA); *L. donovani *(LdCPA),*L. chagasi *(LcCPA) and *L. braziliensis *(LbCPA)); *LiCPA*,*LmjCPA*,*LmxCPA*,*LdCPA*,*LcCPA*,*LbCPA *: CPA genes; *LmxCPC*, *LmxCPB *and LmxCPC, LmxCPB : *L. mexicana *CPC and CPB genes and proteins; DEPC : diethyl pyrocarbonate; DHFR : dihydrofolate reductase; FCS : Foetal Calf Serum; PBS : phosphate buffer saline; SDS : sodium dodecyl sulphate; TAE : Tris-acetate-EDTA buffer; TBE : Tris-borate-EDTA buffer; SDS-PAGE : SDS polyacrylamide gel electrophoresis

## Authors' contributions

JCM, GHC and ANV conceived the project and supervised the experiments. MIJ cloned the JPCM5 line. MIJ, HD and JP created the knockout constructs, made the *Leishmania CPA *mutants and carried out the phenotype analysis. AA helped in the isolation of the *Leishmania *mutants, DCH carried out the dog macrophage studies. MIJ, HD, JP, ANV, GHC and JCM wrote the paper. All authors read and approved the final manuscript.
